# An unsymmetrical dinuclear scandium complex comprising salophen ligands [H_2_salophen = *N*,*N*′-bis­(salicyl­idene)-1,2-phenyl­enedi­amine]

**DOI:** 10.1107/S2056989019000094

**Published:** 2019-01-08

**Authors:** Volker Lorenz, Phil Liebing, Liane Hilfert, Sabine Busse, Frank T. Edelmann

**Affiliations:** a Chemisches Institut der Otto-von-Guericke-Universität Magdeburg, Universitätsplatz 2, 39106 Magdeburg, Germany

**Keywords:** scandium, salophen ligand, Schiff base, dinuclear scandium complex, crystal structure, π–π stacking

## Abstract

Treatment of scandium nitrate tetra­hydrate with the tetra­dentate ligand H_2_salophen [*N*,*N*′-bis­(salicyl­idene)-1,2-phenyl­enedi­amine] afforded the yellow dinuclear complex Sc(NO_3_)_2_(μ-salophen)Sc(salophen)(EtOH) or [Sc_2_(C_20_H_14_N_2_O_2_)_2_(NO_3_)_2_(C_2_H_6_O)] (systematic name: (ethanol-κ*O*)bis­(nitrato-κ^2^ *O*,*O*′){μ-2,2′-[1,2-phenyl­enebis(nitrilo­methanylyl­idene)]diphenolato-κ^4^ *N*,*N*′,*O*,*O*′:κ^2^ *O*,*O*′}{2,2′-[1,2-phenyl­enebis(nitrilo­methanylyl­idene)]diphenolato-κ^4^ *O*,*N*,*N*′,*O*′}discandium). In this compound, one salophen ligand displays a bridging coordination *via* the two oxygen atoms, while the other salophen ligand is attached to only one Sc center. This arrangement is stabilized by a hydrogen-bonded EtOH co-ligand, and by π–π stacking inter­actions between the two salophen ligands.

## Abstract 2

Scandium nitrate tetra­hydrate reacts with H_2_salophen [*N*,*N*′-bis­(salicyl­idene)-1,2-phenyl­enedi­amine] in ethanol to give the unsymmetrical dinuclear complex Sc(NO_3_)_2_(μ-salophen)Sc(salophen)(EtOH).

## Chemical context   

In the coordination chemistry of lanthanides, salen-type Schiff-base ligands such as H_2_salen [*N*,*N*′-bis­(salicyl­idene)-ethyl­enedi­amine] and H_2_salophen [*N*,*N*′-bis­(salicyl­idene)-1,2-phenyl­enedi­amine] are among the best known multidentate ligands. Lanthanide complexes comprising salen-type ligands are of significant inter­est due to their variety of mol­ecular structures (Akine & Nabeshima, 2009 ▸) and their promising magnetic properties (Costes *et al.*, 1998 ▸; Yao *et al.*, 2012 ▸; Pajerowski *et al.*, 2014 ▸) and luminescence properties (Bi *et al.*, 2009 ▸; Li *et al.*, 2013 ▸; Mikhalyova *et al.*, 2014 ▸; Yang *et al.*, 2014 ▸). They also have potential applications in electronic devices (Magadur *et al.*, 2012 ▸) and homogeneous catalysis (Wu *et al.*, 2017 ▸). The first lanthanide–salen and salophen complexes were reported fifty years ago (Dutt & Nag, 1968 ▸). Since then, a variety of inter­esting structures have been reported for such complexes, including mononuclear complexes (Evans *et al.*, 1999 ▸; Yao *et al.*, 2012 ▸), sandwich-like di- and trinuclear species (Chen & Archer, 1994 ▸; Costes *et al.*, 1998 ▸; Camp *et al.*, 2012 ▸; Li *et al.*, 2012 ▸, 2013 ▸; Mikhalyova *et al.*, 2014 ▸), clusters (Zhao *et al.*, 2012 ▸; Pajerowski *et al.*, 2014 ▸) and 3*d*–4*f* heterobimetallic complexes (Condorelli *et al.*, 1975 ▸; Winpenny, 1998 ▸; Sakamoto *et al.*, 2001 ▸; Camp *et al.*, 2017 ▸).

Scandium complexes comprising salen-type Schiff-base ligands are fairly rare, with the majority of such compounds having been reported by Anwander and co-workers (Meermann *et al.*, 2006 ▸, 2009 ▸). Access to these complexes was achieved *via* treatment of the scandium silyl­amide precursor Sc[N(SiHMe_2_)_3_]_3_(THF) with substituted H_2_salen precursors under anaerobic conditions. We report here the straightforward formation and structural characterization of a dinuclear scandium complex comprising salophen ligands using scandium nitrate tetra­hydrate as the starting material. Treatment of a diluted solution of Sc(NO_3_)_3_·4H_2_O in ethanol with an ethano­lic solution of the protonated ligand H_2_salophen (Bonnaire *et al.*, 1981 ▸) resulted in the rapid formation of a yellow precipitate which was identified as the title complex Sc(NO_3_)_2_(μ-salophen)Sc(salophen)(EtOH). The analytically pure material could be isolated in 70% yield. The title compound was fully characterized through the usual set of elemental analysis and spectroscopic methods (IR, NMR, MS). The NMR spectra in DMSO-*d*_6_ solution showed only one set of salophen ^1^H and ^13^C signals, and only one ^45^Sc signal, and consequently the dimeric structure seems to be split into a monomeric species in DMSO. The mass spectrum did not display the mol­ecular ion, but other high-mol­ecular-mass peaks attributable to dimeric species, *e.g.* [*M* − CH_3_]^+^ at *m*/*z* 863, [*M* − EtOH]^+^ at *m*/*z* 843, and [*M* − EtOH − NO_3_]^+^ at *m*/*z* 780.
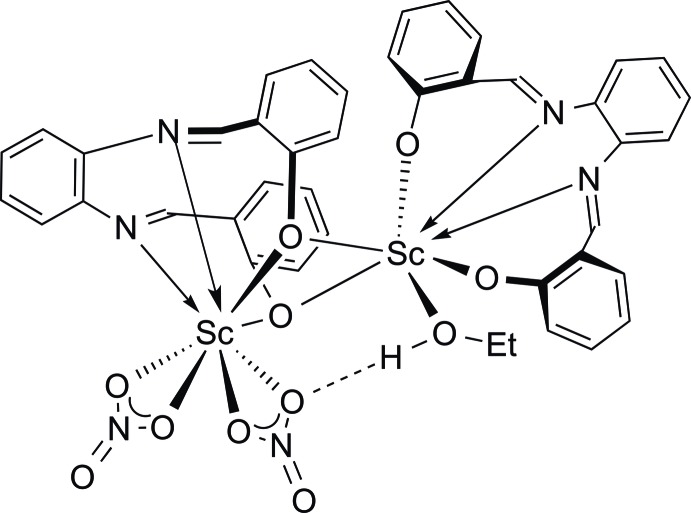


## Structural commentary   

**Figure 1:**
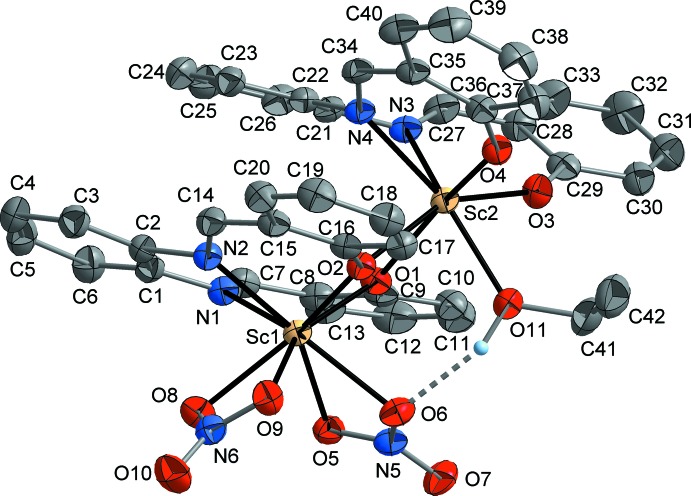
Mol­ecular structure of the title compound in the crystalline state, showing the atom-labelling scheme. Displacement ellipsoids are drawn at the 30% probability level and H atoms attached to C atoms are omitted for clarity.

The asymmetric unit of the title compound recrystallized from ethanol contains two scandium atoms, two nitrate moieties, two salophen ligands, and one EtOH mol­ecule (Fig. 1 ▸). Both Sc atoms are situated in the tetra­dentate coordination pocket of a salophen ligand. Sc1 is coordinatively saturated by two chelating nitrate anions, resulting in a somewhat square-anti­prismatic coordination. Sc2 is connected to the two oxygen atoms of the other Sc(salophen) unit, thus connecting the two parts of the mol­ecule. An irregular seven-coordination of Sc2 is completed by an EtOH ligand. This asymmetrical structure is stabilized by an intra­molecular O—H⋯O hydrogen bond between EtOH and a nitrate ligand [O6⋯O11 2.787 (3) Å, O6⋯H approx. 2.01 Å; Table 1 ▸].

The Sc—O bond lengths within the central Sc_2_O_2_ ring (including O1, O2) are significantly different, and surprisingly the bonds at the seven-coordinated Sc2 [2.214 (2) and 2.342 (2) Å] are longer than those at the octa-coordinated Sc1 [2.062 (2) and 2.110 (2) Å]. The bonds of Sc2 to the terminally coordinated salophene oxygen atoms (O3, O4) are 2.006 (2) and 1.995 (2) Å, respectively. The Sc—N bonds are also slightly longer for Sc2 [2.286 (2) and 2.341 (2) Å] than for Sc1 [2.270 (2) and 2.278 (2) Å]. These values of Sc—N distances are larger than in related scandium–salen complexes (Meermann *et al.*, 2006 ▸, 2009 ▸), reflecting the higher coordination numbers of scandium in the title compound. However, the terminal Sc—O(salicyl­idene) bonds are similar or only marginally elongated as compared to the reference compounds. The Sc—O(nitrate) separations are in the range 2.263 (2)–2.323 (2) Å, resembling the values observed for other scandium–nitrate complexes (*e.g.* Arif *et al.*, 1984 ▸, Cotton *et al.*, 2008 ▸).

The octa-coordinated Sc1 is displaced from the salophene’s N_2_O_2_ coordination plane by 1.091 (1) Å, while the corresponding value for the seven-coordinated Sc2 is only 1.014 (1) Å. Both values are considerably larger than those observed in related complexes (Meermann *et al.*, 2006 ▸, 2009 ▸), which can again be traced back to the higher coordination numbers of scandium. Both salophen ligands deviate markedly from planarity, as the two salicyl­idene arms are twisted out of the particular phenyl­ene-di­amine plane around the C—N single-bonds. The (phenyl­ene)C=C—N=C(imide) torsion angles (which would be 0° in the case of perfect planarity) are 15.7 (4) and 24.6 (4)° for the salophen ligand at Sc1, and 30.0 (4) and 34.7 (4)° for the salophen ligand at Sc2. The corresponding angles between the salicyl­idene C_6_ rings are 12.9 (2)° for Sc1 and 53.5 (1)° for Sc2, being in the same range as in the reference compounds (Meermann *et al.*, 2006 ▸, 2009 ▸). Intra­molecular π–π stacking inter­actions between the two salophen ligands may contribute to the stabilization of the dimeric structure. The two phenyl­ene-di­amine moieties are oriented almost parallel to each other with an angle of 11.8 (1)° between the C_6_ rings, and the closest inter­atomic contact between the rings is 3.401 (4) Å (C2⋯C23). The same is true for the two salicyl­idene moieties, with an angle of 14.4 (1)° and the closest contact being 3.247 (4) Å (C17⋯C35). The remaining two salicyl­idene moieties are not in a proper orientation for efficient π–π stacking [angle between C_6_ rings = 37.1 (2)°].

## Supra­molecular features   

**Figure 2:**
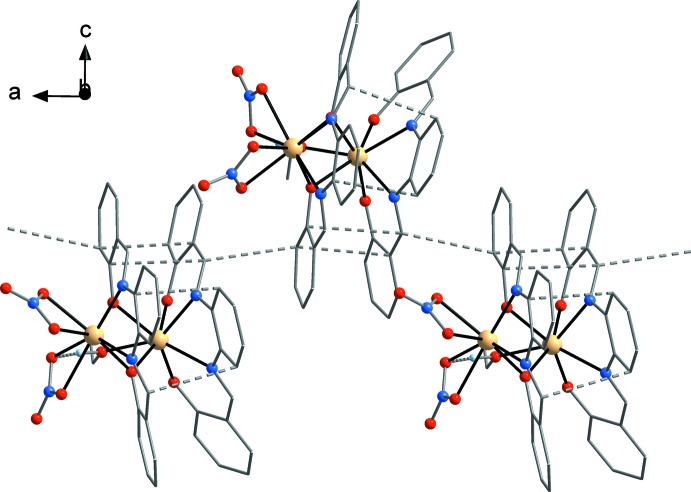
Illustration of intra- and inter­molecular π–π stacking inter­actions. The association of the complex mol­ecules results in a supra­molecular chain structure, which extends along the *a*-axis direction.

The mol­ecules seem to be primarily associated by π–π stacking inter­actions (Fig. 2 ▸). The closest inter­molecular contact is 3.369 (4) Å [C17⋯C34(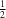 + *x*, *y*, 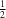 − *z*)] between two salicyl­idene moieties [angle between C_6_ rings of 13.0 (1)°].

## Database survey   

For review articles on rare-earth complexes with salen-type Schiff-base ligands, see: Akine & Nabeshima (2009 ▸); Yang *et al.* (2014 ▸). For review articles on 3*d*–4*f* heteronuclear complexes with polydentate Schiff-base ligands, see: Winpenny (1998 ▸); Sakamoto *et al.* (2001 ▸). For related Sc complexes comprising salen-type Schiff-base ligands, see: Meermann *et al.* (2006 ▸, 2009 ▸).

## Synthesis and crystallization   

0.50 g (1.58 mmol) of H_2_salophen dissolved in *ca* 150 ml of ethanol were added to a solution of 0.63 g (2.08 mmol) Sc(NO_3_)_3_·4H_2_O in 100 ml ethanol at 323 K. After a few minutes the solution became turbid and Sc(NO_3_)_2_(μ-salophen)Sc(salophen)(EtOH) precipitated as a microcrystalline yellow solid. Yield: 0.5 g (70%). Recrystallization from hot ethanol afforded yellow, plate-like single crystals. Decomp. 443 K. Analysis calculated for C_42_H_34_N_6_O_11_Sc_2_ (*M* = 888.68 g mol^−1^): C 56.77, H 3.86, N 9.46; found: C 56.36, H 3.95, N 9.81%.

**^1^H NMR** (400.1 MHz, DMSO-*d*_6_, 294 K): δ = 8.74 (*s*, 4H, *H*C=N), 7.71–7.68 (*m*, 4H, *m*-C_6_ *H*_4_N), 7.57 (*d*, 4H, *o*-C_6_ *H*_4_C), 7.46–7.43 (*m*, 4H, *o*-C_6_ *H*_4_N), 7.38 (*t*, 4H, *m*-C_6_ *H*_4_C), 6.73 (*d*, 4H, *o*-C_6_ *H*_4_O), 6.71 (*t*, 4H, *m*-C_6_ *H*_4_O) ppm; C*H*_3_C*H*_2_O*H* not observed. **^13^C NMR** (100.6 MHz, DMSO-*d*_6_, 294 K): δ = 166.1 (O—*C*_6_H_4_), 161.9 (H*C*=N), 144.2 (N—*C*_6_H_4_), 135.1 (*o*-*C*_6_H_4_C, *m*-*C*_6_H_4_C), 128.0 (*o*-*C*_6_H_4_N), 122.5 (C-*C*_6_H_4_), 120.3 (*o*-*C*_6_H_4_O), 118.3 (*m*-*C*_6_H_4_N), 115.5 (*m*-*C*_6_H_4_O) ppm; *C*H_3_ *C*H_2_OH not observed. **^45^Sc NMR** (97.2 MHz, DMSO-*d*_6_, 294 KC): δ = 49.8 ppm.

**IR** (ATR): *ν* = 3426*w*, 3058*w*, 3026*w*, 2973*w*, 1609*vs*, 1580*m*, 1540*m*, 1526*s*, 1472*s*, 1443*m*, 1380*m*, 1348*w*, 1300*s*, 1276*s*, 1236*m*, 1193*m*, 1150*m*, 1123*m*, 1032*w*, 1021*w*, 984*w*, 946*w*, 920*m*, 864*w*, 851*w*, 802*m*, 747*vs*, 729*s*, 699*w*, 667*w*, 641*w*, 606*m*, 583*w*, 564*m*, 531*s*, 513*w*, 486*m*, 470*w*, 450*m*, 400*m*, 378*v*s, 353*m*, 306*vs*, 281*vs*, 231*m*, 169*w*, 157*w*, 137*w*, 118w, 107*w*, 91*w*, 77*w*, 68w, 61*w*, 54*w* cm^−1^.

**MS** (70 eV): *m*/*z* = 863 (1%) [*M* − CH_3_]^+^, 843 (<1%) [*M* − EtOH]^+^, 780 (2%) [*M* − EtOH − NO_3_]^+^, 733 (4%), 705 (1%), 662 (65%) [*M* − EtOH − NO_3_ – NC_6_H_4_O]^+^, 647 (70%), 580 (5%), 568 (27%), 555 (8%), 506 (100%), 480 (42%).

## Refinement   

Crystal data, data collection and structure refinement details are summarized in Table 2 ▸. H atoms attached to C atoms were fixed geometrically and refined using a riding model. All C—H distances within the salophen ligands were constrained to 0.95 Å. For the EtOH ligand, the C—H distances within the CH_2_ group were constrained to 0.99 Å, the C—H distances within the CH_3_ group were constrained to 0.98 Å, and the CH_3_ group was allowed to rotate freely around the C–C vector. The oxygen-bound EtOH hydrogen atom was located in the difference-Fourier map and refined freely, the corresponding O—H distance was restrained to 0.84 (2) Å. The *U*_iso_(H) values were set at 1.2*U*_eq_(*X*) (*X* = C, O). The reflections 020 and 021 disagreed strongly with the structural model and were therefore omitted from the refinement.

## Supplementary Material

Crystal structure: contains datablock(s) I. DOI: 10.1107/S2056989019000094/zl2746sup1.cif

Structure factors: contains datablock(s) I. DOI: 10.1107/S2056989019000094/zl2746Isup2.hkl

CCDC reference: 1880860

Additional supporting information:  crystallographic information; 3D view; checkCIF report

## Figures and Tables

**Table 1:** Hydrogen-bond geometry (Å, °)

*D*—H⋯*A*	*D*—H	H⋯*A*	*D*⋯*A*	*D*—H⋯*A*
O11—H29⋯O6	0.83 (2)	2.01 (2)	2.787 (3)	156 (3)

**Table 2:** Experimental details

Crystal data	
Chemical formula	[Sc_2_(C_42_H_34_N_6_O_11_)]
*M*_r_	888.67
Crystal system, space group	Orthorhombic, *P* *b* *c* *a*
Temperature (K)	153
*a*, *b*, *c* (Å)	13.6092 (3), 21.5880 (7), 26.5297 (7)
*V* (Å^3^)	7794.3 (4)
*Z*	8
Radiation type	Mo *K*α
μ (mm^−1^)	0.42
Crystal size (mm)	0.32 × 0.13 × 0.09

Data collection	
Diffractometer	STOE IPDS 2T
No. of measured, independent and observed [*I* > 2σ(*I*)] reflections	25118, 6849, 5105
*R*_int_	0.064
(sin θ/λ)_max_ (Å^−1^)	0.595

Refinement	
*R*[*F* ^2^ > 2σ(*F* ^2^)], *wR*(*F* ^2^), *S*	0.045, 0.098, 1.04
No. of reflections	6849
No. of parameters	555
No. of restraints	1
H-atom treatment	H atoms treated by a mixture of independent and constrained refinement
Δρ_max_, Δρ_min_ (e Å^−3^)	0.25, −0.34

Computer programs: *X-AREA* and *X-RED32* (Stoe & Cie, 2002 ▸), *SIR97* (Altomare *et al.*, 1999 ▸), *SHELXL2018/3* (Sheldrick, 2015 ▸), *DIAMOND* (Brandenburg, 1999 ▸) and *publCIF* (Westrip, 2010 ▸).

## supplementary crystallographic information

### Crystal data

**Table:**

[Sc_2_(C_20_H_14_N_2_O_2_)_2_(NO_3_)_2_(C_2_H_6_O)]	*D*_x_ = 1.515 Mg m^−^^3^
*M**_r_* = 888.67	Mo *K*α radiation, λ = 0.71073 Å
Orthorhombic, *P**b**c**a*	Cell parameters from 25274 reflections
*a* = 13.6092 (3) Å	θ = 1.9–25.1°
*b* = 21.5880 (7) Å	µ = 0.42 mm^−^^1^
*c* = 26.5297 (7) Å	*T* = 153 K
*V* = 7794.3 (4) Å^3^	Plate, yellow
*Z* = 8	0.32 × 0.13 × 0.09 mm
*F*(000) = 3664	

### Data collection

**Table:**

STOE IPDS 2T diffractometer	5105 reflections with *I* > 2σ(*I*)
Radiation source: fine-focus sealed tube	*R*_int_ = 0.064
Detector resolution: 6.67 pixels mm^-1^	θ_max_ = 25.0°, θ_min_ = 1.9°
area detector scans	*h* = −16→13
25118 measured reflections	*k* = −25→25
6849 independent reflections	*l* = −27→31

### Refinement

**Table:**

Refinement on *F*^2^	Secondary atom site location: difference Fourier map
Least-squares matrix: full	Hydrogen site location: mixed
*R*[*F*^2^ > 2σ(*F*^2^)] = 0.045	H atoms treated by a mixture of independent and constrained refinement
*wR*(*F*^2^) = 0.098	*w* = 1/[σ^2^(*F*_o_^2^) + (0.0448*P*)^2^ + 1.8222*P*] where *P* = (*F*_o_^2^ + 2*F*_c_^2^)/3
*S* = 1.04	(Δ/σ)_max_ = 0.001
6849 reflections	Δρ_max_ = 0.25 e Å^−^^3^
555 parameters	Δρ_min_ = −0.34 e Å^−^^3^
1 restraint	Extinction correction: *SHELXL2018/3* (Sheldrick, 2015), Fc^*^=kFc[1+0.001xFc^2^λ^3^/sin(2θ)]^-1/4^
Primary atom site location: heavy-atom method	Extinction coefficient: 0.00110 (14)

### Special details

**Table:**

Geometry. All esds (except the esd in the dihedral angle between two l.s. planes) are estimated using the full covariance matrix. The cell esds are taken into account individually in the estimation of esds in distances, angles and torsion angles; correlations between esds in cell parameters are only used when they are defined by crystal symmetry. An approximate (isotropic) treatment of cell esds is used for estimating esds involving l.s. planes.

### Fractional atomic coordinates and isotropic or equivalent isotropic displacement parameters (Å^2^)

**Table:**

	*x*	*y*	*z*	*U*_iso_*/*U*_eq_	
C1	0.4169 (2)	0.37468 (12)	0.37720 (10)	0.0288 (6)	
C2	0.4285 (2)	0.36467 (12)	0.32549 (10)	0.0286 (6)	
C3	0.4074 (2)	0.41236 (13)	0.29195 (11)	0.0374 (7)	
H1	0.415046	0.405955	0.256739	0.045*	
C4	0.3755 (2)	0.46863 (14)	0.30958 (12)	0.0417 (7)	
H2	0.359832	0.500726	0.286441	0.050*	
C5	0.3661 (3)	0.47893 (13)	0.36070 (12)	0.0440 (8)	
H3	0.343986	0.518025	0.372627	0.053*	
C6	0.3887 (2)	0.43240 (13)	0.39456 (12)	0.0395 (7)	
H4	0.384852	0.440072	0.429763	0.047*	
C7	0.3910 (2)	0.31818 (13)	0.45144 (10)	0.0311 (6)	
H5	0.354354	0.353106	0.462447	0.037*	
C8	0.3950 (2)	0.26510 (13)	0.48419 (10)	0.0305 (6)	
C9	0.4137 (2)	0.20494 (13)	0.46694 (10)	0.0293 (6)	
C10	0.4117 (2)	0.15587 (14)	0.50090 (10)	0.0362 (7)	
H6	0.426733	0.115071	0.489928	0.043*	
C11	0.3876 (3)	0.16692 (16)	0.55097 (11)	0.0462 (8)	
H7	0.384366	0.133093	0.573820	0.055*	
C12	0.3682 (3)	0.22552 (17)	0.56815 (10)	0.0454 (8)	
H8	0.352461	0.232186	0.602605	0.054*	
C13	0.3718 (2)	0.27431 (15)	0.53541 (10)	0.0384 (7)	
H9	0.358442	0.314954	0.547320	0.046*	
C14	0.4470 (2)	0.28648 (13)	0.26559 (10)	0.0310 (6)	
H10	0.418676	0.315312	0.242784	0.037*	
C15	0.4732 (2)	0.22675 (12)	0.24603 (10)	0.0291 (6)	
C16	0.4917 (2)	0.17455 (12)	0.27593 (9)	0.0254 (6)	
C17	0.5164 (2)	0.11931 (13)	0.25251 (10)	0.0305 (6)	
H11	0.531690	0.083924	0.272314	0.037*	
C18	0.5190 (2)	0.11538 (13)	0.20037 (11)	0.0351 (7)	
H12	0.535121	0.077002	0.184917	0.042*	
C19	0.4988 (2)	0.16611 (14)	0.17044 (10)	0.0369 (7)	
H13	0.500778	0.162894	0.134746	0.044*	
C20	0.4758 (2)	0.22097 (13)	0.19312 (10)	0.0348 (7)	
H14	0.461175	0.256021	0.172809	0.042*	
C21	0.2072 (2)	0.22444 (12)	0.38836 (10)	0.0292 (6)	
C22	0.2308 (2)	0.22964 (12)	0.33720 (10)	0.0278 (6)	
C23	0.2145 (2)	0.28503 (13)	0.31215 (11)	0.0347 (6)	
H15	0.229900	0.288685	0.277355	0.042*	
C24	0.1756 (2)	0.33495 (14)	0.33805 (12)	0.0414 (7)	
H16	0.165563	0.373207	0.321119	0.050*	
C25	0.1513 (2)	0.32942 (15)	0.38855 (12)	0.0439 (8)	
H17	0.123577	0.363688	0.405934	0.053*	
C26	0.1671 (2)	0.27442 (13)	0.41373 (11)	0.0368 (7)	
H18	0.150594	0.270862	0.448400	0.044*	
C27	0.1757 (2)	0.14652 (14)	0.44698 (10)	0.0347 (7)	
H19	0.123756	0.172725	0.457844	0.042*	
C28	0.1883 (2)	0.08875 (14)	0.47336 (10)	0.0358 (7)	
C29	0.2627 (2)	0.04610 (13)	0.45997 (10)	0.0318 (6)	
C30	0.2688 (2)	−0.00922 (13)	0.48758 (10)	0.0371 (7)	
H20	0.318190	−0.038658	0.479358	0.044*	
C31	0.2046 (3)	−0.02153 (15)	0.52636 (11)	0.0448 (8)	
H21	0.210072	−0.059403	0.544379	0.054*	
C32	0.1325 (3)	0.02028 (18)	0.53944 (12)	0.0558 (9)	
H22	0.088460	0.011353	0.566272	0.067*	
C33	0.1248 (3)	0.07493 (17)	0.51331 (12)	0.0503 (9)	
H23	0.075452	0.103930	0.522519	0.060*	
C34	0.2507 (2)	0.16197 (13)	0.26938 (10)	0.0302 (6)	
H24	0.213717	0.191577	0.250913	0.036*	
C35	0.2793 (2)	0.10654 (12)	0.24355 (10)	0.0299 (6)	
C36	0.3223 (2)	0.05556 (12)	0.26821 (10)	0.0300 (6)	
C37	0.3414 (2)	0.00209 (13)	0.24052 (11)	0.0375 (7)	
H25	0.368266	−0.033093	0.257046	0.045*	
C38	0.3221 (3)	−0.00068 (15)	0.18940 (11)	0.0453 (8)	
H26	0.337343	−0.037128	0.170926	0.054*	
C39	0.2804 (3)	0.04985 (16)	0.16510 (11)	0.0466 (8)	
H27	0.266941	0.047973	0.129993	0.056*	
C40	0.2586 (2)	0.10217 (15)	0.19168 (10)	0.0400 (7)	
H28	0.229035	0.136271	0.174894	0.048*	
C41	0.5291 (3)	0.00620 (14)	0.39612 (13)	0.0490 (8)	
H31	0.474832	−0.011480	0.416354	0.059*	
H30	0.590119	0.003390	0.416346	0.059*	
C42	0.5411 (3)	−0.03072 (16)	0.34884 (16)	0.0658 (12)	
H32	0.552749	−0.074255	0.357452	0.079*	
H34	0.597145	−0.014731	0.329630	0.079*	
H33	0.481301	−0.027384	0.328455	0.079*	
N1	0.43390 (17)	0.32154 (10)	0.40802 (8)	0.0285 (5)	
N2	0.45891 (17)	0.30419 (9)	0.31149 (8)	0.0267 (5)	
N3	0.22859 (17)	0.16566 (10)	0.41003 (8)	0.0291 (5)	
N4	0.27121 (16)	0.17499 (10)	0.31579 (8)	0.0266 (5)	
N5	0.64150 (17)	0.19007 (10)	0.44454 (8)	0.0306 (5)	
N6	0.68564 (18)	0.30939 (11)	0.33592 (8)	0.0318 (5)	
O1	0.43324 (14)	0.19366 (8)	0.41759 (6)	0.0270 (4)	
O2	0.48202 (14)	0.17742 (8)	0.32666 (6)	0.0258 (4)	
O3	0.32397 (15)	0.05685 (8)	0.42285 (7)	0.0329 (4)	
O4	0.34377 (15)	0.05783 (8)	0.31673 (7)	0.0311 (4)	
O5	0.61104 (15)	0.24485 (8)	0.45084 (7)	0.0337 (4)	
O6	0.63584 (15)	0.16986 (9)	0.39927 (6)	0.0326 (4)	
O7	0.67244 (17)	0.15794 (10)	0.47845 (7)	0.0424 (5)	
O8	0.63219 (15)	0.33161 (8)	0.37050 (7)	0.0346 (4)	
O9	0.66019 (15)	0.25579 (9)	0.32055 (7)	0.0348 (5)	
O10	0.75629 (17)	0.33665 (10)	0.31869 (9)	0.0485 (6)	
O11	0.50835 (16)	0.07027 (9)	0.38540 (8)	0.0379 (5)	
H29	0.5584 (18)	0.0918 (14)	0.3877 (13)	0.046*	
Sc1	0.53071 (4)	0.24711 (2)	0.37359 (2)	0.02440 (13)	
Sc2	0.36511 (4)	0.11983 (2)	0.37139 (2)	0.02536 (13)	

### Atomic displacement parameters (Å^2^)

**Table:**

	*U*^11^	*U*^22^	*U*^33^	*U*^12^	*U*^13^	*U*^23^
C1	0.0250 (15)	0.0306 (13)	0.0307 (14)	0.0029 (11)	0.0024 (11)	0.0012 (11)
C2	0.0274 (15)	0.0263 (13)	0.0320 (15)	0.0003 (11)	−0.0023 (12)	0.0002 (11)
C3	0.0428 (19)	0.0350 (15)	0.0345 (15)	−0.0001 (13)	−0.0032 (13)	0.0028 (12)
C4	0.045 (2)	0.0323 (15)	0.0482 (18)	0.0057 (14)	−0.0044 (15)	0.0063 (13)
C5	0.048 (2)	0.0310 (15)	0.054 (2)	0.0089 (14)	0.0088 (16)	0.0004 (13)
C6	0.0418 (19)	0.0371 (16)	0.0396 (16)	0.0052 (14)	0.0095 (14)	−0.0028 (13)
C7	0.0298 (16)	0.0362 (15)	0.0272 (14)	−0.0008 (12)	0.0019 (11)	−0.0057 (11)
C8	0.0276 (15)	0.0431 (16)	0.0209 (13)	−0.0002 (12)	0.0021 (11)	−0.0012 (11)
C9	0.0239 (15)	0.0417 (15)	0.0222 (13)	−0.0042 (12)	−0.0010 (11)	0.0007 (11)
C10	0.0417 (18)	0.0431 (16)	0.0238 (14)	−0.0059 (14)	−0.0053 (12)	0.0049 (12)
C11	0.051 (2)	0.060 (2)	0.0274 (15)	−0.0099 (17)	−0.0071 (14)	0.0116 (14)
C12	0.044 (2)	0.074 (2)	0.0184 (14)	−0.0079 (17)	−0.0006 (13)	0.0010 (14)
C13	0.0328 (17)	0.0550 (18)	0.0273 (14)	0.0013 (14)	0.0007 (13)	−0.0050 (13)
C14	0.0321 (17)	0.0355 (15)	0.0254 (14)	−0.0013 (12)	−0.0019 (12)	0.0051 (11)
C15	0.0291 (15)	0.0360 (14)	0.0220 (13)	−0.0022 (12)	−0.0014 (11)	−0.0017 (11)
C16	0.0219 (14)	0.0339 (14)	0.0204 (12)	−0.0026 (11)	0.0012 (10)	−0.0022 (10)
C17	0.0294 (16)	0.0335 (14)	0.0287 (14)	0.0018 (12)	0.0000 (12)	−0.0017 (11)
C18	0.0356 (17)	0.0401 (15)	0.0294 (15)	0.0012 (13)	0.0057 (13)	−0.0074 (12)
C19	0.0436 (19)	0.0505 (17)	0.0165 (13)	−0.0041 (14)	0.0035 (12)	−0.0044 (12)
C20	0.0418 (18)	0.0396 (15)	0.0230 (14)	−0.0052 (14)	−0.0024 (12)	0.0022 (12)
C21	0.0272 (15)	0.0340 (14)	0.0265 (13)	0.0028 (12)	−0.0053 (11)	−0.0017 (11)
C22	0.0225 (14)	0.0316 (14)	0.0292 (14)	−0.0002 (11)	−0.0025 (11)	−0.0013 (11)
C23	0.0320 (16)	0.0375 (15)	0.0345 (15)	−0.0009 (13)	−0.0021 (13)	0.0064 (12)
C24	0.0364 (18)	0.0344 (16)	0.0536 (19)	0.0055 (14)	−0.0031 (15)	0.0023 (14)
C25	0.041 (2)	0.0436 (17)	0.0474 (18)	0.0086 (15)	−0.0021 (15)	−0.0104 (14)
C26	0.0377 (18)	0.0438 (16)	0.0289 (14)	0.0060 (14)	−0.0027 (13)	−0.0073 (12)
C27	0.0330 (17)	0.0464 (16)	0.0247 (14)	0.0037 (13)	0.0002 (12)	−0.0007 (12)
C28	0.0364 (18)	0.0461 (16)	0.0249 (14)	−0.0017 (14)	0.0008 (12)	0.0031 (12)
C29	0.0363 (17)	0.0399 (15)	0.0194 (12)	−0.0061 (13)	−0.0051 (12)	−0.0013 (11)
C30	0.049 (2)	0.0366 (15)	0.0257 (14)	−0.0030 (14)	−0.0060 (13)	0.0014 (12)
C31	0.056 (2)	0.0489 (18)	0.0290 (16)	−0.0093 (17)	−0.0032 (15)	0.0075 (13)
C32	0.056 (2)	0.076 (2)	0.0347 (17)	−0.003 (2)	0.0145 (17)	0.0181 (16)
C33	0.046 (2)	0.071 (2)	0.0337 (17)	0.0087 (18)	0.0116 (15)	0.0118 (16)
C34	0.0252 (15)	0.0388 (15)	0.0264 (14)	−0.0005 (12)	−0.0041 (11)	0.0041 (11)
C35	0.0274 (15)	0.0403 (15)	0.0220 (13)	−0.0012 (12)	−0.0025 (11)	−0.0004 (11)
C36	0.0339 (16)	0.0360 (15)	0.0202 (13)	−0.0057 (12)	−0.0014 (11)	−0.0005 (11)
C37	0.047 (2)	0.0350 (15)	0.0306 (15)	−0.0037 (14)	0.0006 (13)	−0.0029 (12)
C38	0.058 (2)	0.0470 (18)	0.0312 (16)	−0.0045 (16)	0.0019 (15)	−0.0129 (14)
C39	0.053 (2)	0.062 (2)	0.0243 (15)	0.0008 (17)	−0.0046 (14)	−0.0096 (14)
C40	0.0403 (19)	0.0549 (18)	0.0248 (14)	0.0022 (15)	−0.0063 (13)	−0.0018 (13)
C41	0.047 (2)	0.0432 (17)	0.057 (2)	0.0106 (16)	0.0011 (17)	0.0158 (16)
C42	0.085 (3)	0.0386 (19)	0.074 (3)	0.0079 (19)	−0.026 (2)	−0.0036 (18)
N1	0.0287 (13)	0.0332 (12)	0.0237 (11)	0.0005 (10)	0.0010 (9)	−0.0003 (9)
N2	0.0301 (13)	0.0277 (11)	0.0223 (11)	0.0005 (10)	−0.0004 (9)	−0.0009 (9)
N3	0.0296 (13)	0.0379 (12)	0.0197 (11)	−0.0004 (10)	−0.0032 (10)	−0.0008 (9)
N4	0.0245 (12)	0.0326 (11)	0.0227 (11)	0.0000 (10)	−0.0007 (9)	0.0003 (9)
N5	0.0277 (13)	0.0382 (13)	0.0258 (12)	0.0006 (11)	−0.0012 (10)	−0.0020 (10)
N6	0.0302 (14)	0.0364 (13)	0.0288 (12)	0.0012 (11)	0.0008 (10)	0.0029 (10)
O1	0.0303 (10)	0.0331 (10)	0.0175 (8)	−0.0032 (8)	0.0000 (7)	−0.0019 (7)
O2	0.0297 (11)	0.0306 (9)	0.0172 (8)	−0.0004 (8)	−0.0009 (7)	0.0004 (7)
O3	0.0383 (12)	0.0361 (10)	0.0241 (9)	0.0006 (9)	0.0052 (9)	0.0038 (8)
O4	0.0381 (12)	0.0320 (10)	0.0232 (9)	−0.0017 (8)	−0.0046 (8)	0.0004 (7)
O5	0.0335 (11)	0.0336 (10)	0.0342 (10)	0.0013 (9)	−0.0062 (9)	−0.0044 (8)
O6	0.0329 (11)	0.0433 (11)	0.0217 (9)	0.0056 (9)	−0.0023 (8)	−0.0028 (8)
O7	0.0509 (14)	0.0479 (12)	0.0283 (10)	0.0061 (10)	−0.0109 (10)	0.0092 (9)
O8	0.0333 (11)	0.0379 (10)	0.0327 (10)	−0.0032 (9)	0.0057 (9)	−0.0067 (9)
O9	0.0372 (12)	0.0314 (10)	0.0358 (11)	0.0003 (9)	0.0089 (9)	−0.0044 (8)
O10	0.0415 (14)	0.0516 (13)	0.0524 (13)	−0.0117 (11)	0.0150 (11)	0.0058 (10)
O11	0.0334 (12)	0.0318 (10)	0.0486 (12)	0.0013 (9)	−0.0052 (10)	0.0062 (9)
Sc1	0.0253 (3)	0.0282 (2)	0.0197 (2)	0.0010 (2)	−0.0002 (2)	−0.0008 (2)
Sc2	0.0274 (3)	0.0291 (2)	0.0197 (2)	0.0004 (2)	−0.0019 (2)	0.0002 (2)

### Geometric parameters (Å, º)

**Table:**

C1—C6	1.383 (4)	C28—C29	1.414 (4)
C1—C2	1.398 (4)	C29—O3	1.311 (3)
C1—N1	1.428 (3)	C29—C30	1.404 (4)
C2—C3	1.391 (4)	C30—C31	1.376 (4)
C2—N2	1.419 (3)	C30—H20	0.9500
C3—C4	1.372 (4)	C31—C32	1.378 (5)
C3—H1	0.9500	C31—H21	0.9500
C4—C5	1.380 (4)	C32—C33	1.372 (5)
C4—H2	0.9500	C32—H22	0.9500
C5—C6	1.382 (4)	C33—H23	0.9500
C5—H3	0.9500	C34—N4	1.293 (3)
C6—H4	0.9500	C34—C35	1.433 (4)
C7—N1	1.293 (3)	C34—H24	0.9500
C7—C8	1.439 (4)	C35—C40	1.408 (4)
C7—H5	0.9500	C35—C36	1.408 (4)
C8—C9	1.400 (4)	C36—O4	1.321 (3)
C8—C13	1.409 (4)	C36—C37	1.393 (4)
C9—O1	1.358 (3)	C37—C38	1.383 (4)
C9—C10	1.391 (4)	C37—H25	0.9500
C10—C11	1.389 (4)	C38—C39	1.389 (5)
C10—H6	0.9500	C38—H26	0.9500
C11—C12	1.370 (5)	C39—C40	1.364 (4)
C11—H7	0.9500	C39—H27	0.9500
C12—C13	1.366 (4)	C40—H28	0.9500
C12—H8	0.9500	C41—O11	1.440 (3)
C13—H9	0.9500	C41—C42	1.495 (5)
C14—N2	1.287 (3)	C41—H31	0.9900
C14—C15	1.435 (4)	C41—H30	0.9900
C14—H10	0.9500	C42—H32	0.9800
C15—C16	1.401 (4)	C42—H34	0.9800
C15—C20	1.410 (4)	C42—H33	0.9800
C16—O2	1.354 (3)	N1—Sc1	2.270 (2)
C16—C17	1.386 (4)	N2—Sc1	2.278 (2)
C17—C18	1.386 (4)	N3—Sc2	2.341 (2)
C17—H11	0.9500	N4—Sc2	2.286 (2)
C18—C19	1.380 (4)	N5—O7	1.212 (3)
C18—H12	0.9500	N5—O5	1.264 (3)
C19—C20	1.365 (4)	N5—O6	1.280 (3)
C19—H13	0.9500	N5—Sc1	2.708 (2)
C20—H14	0.9500	N6—O10	1.216 (3)
C21—C26	1.384 (4)	N6—O8	1.265 (3)
C21—C22	1.399 (4)	N6—O9	1.275 (3)
C21—N3	1.423 (3)	N6—Sc1	2.693 (2)
C22—C23	1.386 (4)	O1—Sc1	2.1103 (18)
C22—N4	1.420 (3)	O1—Sc2	2.2139 (18)
C23—C24	1.383 (4)	O2—Sc1	2.0622 (18)
C23—H15	0.9500	O2—Sc2	2.3420 (18)
C24—C25	1.385 (5)	O3—Sc2	2.0065 (18)
C24—H16	0.9500	O4—Sc2	1.9949 (18)
C25—C26	1.379 (4)	O5—Sc1	2.3233 (19)
C25—H17	0.9500	O6—Sc1	2.300 (2)
C26—H18	0.9500	O8—Sc1	2.289 (2)
C27—N3	1.285 (4)	O9—Sc1	2.263 (2)
C27—C28	1.440 (4)	O11—Sc2	2.255 (2)
C27—H19	0.9500	O11—H29	0.827 (18)
C28—C33	1.400 (4)	Sc1—Sc2	3.5541 (7)
			
C6—C1—C2	119.8 (3)	C41—C42—H34	109.5
C6—C1—N1	125.3 (2)	H32—C42—H34	109.5
C2—C1—N1	114.8 (2)	C41—C42—H33	109.5
C3—C2—C1	119.3 (2)	H32—C42—H33	109.5
C3—C2—N2	125.0 (2)	H34—C42—H33	109.5
C1—C2—N2	115.6 (2)	C7—N1—C1	118.8 (2)
C4—C3—C2	120.2 (3)	C7—N1—Sc1	125.50 (19)
C4—C3—H1	119.9	C1—N1—Sc1	115.62 (16)
C2—C3—H1	119.9	C14—N2—C2	119.0 (2)
C3—C4—C5	120.5 (3)	C14—N2—Sc1	125.29 (18)
C3—C4—H2	119.8	C2—N2—Sc1	115.69 (16)
C5—C4—H2	119.8	C27—N3—C21	118.7 (2)
C4—C5—C6	120.0 (3)	C27—N3—Sc2	130.0 (2)
C4—C5—H3	120.0	C21—N3—Sc2	111.25 (17)
C6—C5—H3	120.0	C34—N4—C22	118.6 (2)
C5—C6—C1	120.0 (3)	C34—N4—Sc2	128.43 (19)
C5—C6—H4	120.0	C22—N4—Sc2	113.00 (15)
C1—C6—H4	120.0	O7—N5—O5	123.5 (2)
N1—C7—C8	124.4 (3)	O7—N5—O6	121.5 (2)
N1—C7—H5	117.8	O5—N5—O6	115.0 (2)
C8—C7—H5	117.8	O7—N5—Sc1	165.9 (2)
C9—C8—C13	119.1 (3)	O5—N5—Sc1	58.94 (12)
C9—C8—C7	123.2 (2)	O6—N5—Sc1	57.95 (12)
C13—C8—C7	117.5 (3)	O10—N6—O8	122.9 (2)
O1—C9—C10	119.5 (2)	O10—N6—O9	122.2 (2)
O1—C9—C8	121.1 (2)	O8—N6—O9	114.8 (2)
C10—C9—C8	119.4 (2)	O10—N6—Sc1	179.0 (2)
C11—C10—C9	119.6 (3)	O8—N6—Sc1	58.00 (13)
C11—C10—H6	120.2	O9—N6—Sc1	56.83 (12)
C9—C10—H6	120.2	C9—O1—Sc1	123.94 (16)
C12—C11—C10	121.5 (3)	C9—O1—Sc2	125.50 (16)
C12—C11—H7	119.3	Sc1—O1—Sc2	110.53 (7)
C10—C11—H7	119.3	C16—O2—Sc1	127.04 (16)
C13—C12—C11	119.6 (3)	C16—O2—Sc2	123.06 (15)
C13—C12—H8	120.2	Sc1—O2—Sc2	107.44 (7)
C11—C12—H8	120.2	C29—O3—Sc2	143.93 (18)
C12—C13—C8	120.8 (3)	C36—O4—Sc2	139.96 (17)
C12—C13—H9	119.6	N5—O5—Sc1	93.28 (14)
C8—C13—H9	119.6	N5—O6—Sc1	93.91 (14)
N2—C14—C15	125.3 (3)	N6—O8—Sc1	94.06 (15)
N2—C14—H10	117.3	N6—O9—Sc1	95.04 (14)
C15—C14—H10	117.3	C41—O11—Sc2	131.2 (2)
C16—C15—C20	119.2 (2)	C41—O11—H29	111 (2)
C16—C15—C14	124.2 (2)	Sc2—O11—H29	117 (2)
C20—C15—C14	116.5 (2)	O2—Sc1—O1	74.52 (7)
O2—C16—C17	120.6 (2)	O2—Sc1—O9	86.29 (7)
O2—C16—C15	120.6 (2)	O1—Sc1—O9	151.49 (7)
C17—C16—C15	118.8 (2)	O2—Sc1—N1	124.95 (8)
C16—C17—C18	120.4 (3)	O1—Sc1—N1	78.44 (8)
C16—C17—H11	119.8	O9—Sc1—N1	130.06 (8)
C18—C17—H11	119.8	O2—Sc1—N2	79.64 (7)
C19—C18—C17	121.4 (3)	O1—Sc1—N2	115.23 (8)
C19—C18—H12	119.3	O9—Sc1—N2	80.76 (8)
C17—C18—H12	119.3	N1—Sc1—N2	70.06 (8)
C20—C19—C18	118.7 (3)	O2—Sc1—O8	138.89 (7)
C20—C19—H13	120.6	O1—Sc1—O8	146.58 (7)
C18—C19—H13	120.6	O9—Sc1—O8	56.08 (7)
C19—C20—C15	121.4 (3)	N1—Sc1—O8	78.49 (8)
C19—C20—H14	119.3	N2—Sc1—O8	78.57 (8)
C15—C20—H14	119.3	O2—Sc1—O6	81.36 (7)
C26—C21—C22	120.0 (3)	O1—Sc1—O6	80.26 (7)
C26—C21—N3	125.4 (2)	O9—Sc1—O6	76.10 (7)
C22—C21—N3	114.6 (2)	N1—Sc1—O6	139.03 (7)
C23—C22—C21	119.8 (3)	N2—Sc1—O6	150.84 (7)
C23—C22—N4	125.9 (2)	O8—Sc1—O6	102.30 (7)
C21—C22—N4	114.2 (2)	O2—Sc1—O5	131.95 (7)
C24—C23—C22	119.7 (3)	O1—Sc1—O5	78.25 (7)
C24—C23—H15	120.1	O9—Sc1—O5	100.59 (8)
C22—C23—H15	120.1	N1—Sc1—O5	86.16 (7)
C23—C24—C25	120.3 (3)	N2—Sc1—O5	148.35 (7)
C23—C24—H16	119.9	O8—Sc1—O5	76.38 (7)
C25—C24—H16	119.9	O6—Sc1—O5	55.31 (6)
C26—C25—C24	120.4 (3)	O2—Sc1—N6	113.07 (7)
C26—C25—H17	119.8	O1—Sc1—N6	166.03 (7)
C24—C25—H17	119.8	O9—Sc1—N6	28.14 (7)
C25—C26—C21	119.8 (3)	N1—Sc1—N6	104.50 (8)
C25—C26—H18	120.1	N2—Sc1—N6	78.31 (8)
C21—C26—H18	120.1	O8—Sc1—N6	27.94 (7)
N3—C27—C28	125.6 (3)	O6—Sc1—N6	89.13 (7)
N3—C27—H19	117.2	O5—Sc1—N6	88.25 (7)
C28—C27—H19	117.2	O2—Sc1—N5	105.47 (7)
C33—C28—C29	119.6 (3)	O1—Sc1—N5	73.55 (7)
C33—C28—C27	118.6 (3)	O9—Sc1—N5	92.08 (7)
C29—C28—C27	121.8 (3)	N1—Sc1—N5	111.43 (7)
O3—C29—C30	120.3 (3)	N2—Sc1—N5	171.01 (8)
O3—C29—C28	121.9 (2)	O8—Sc1—N5	92.95 (7)
C30—C29—C28	117.8 (3)	O6—Sc1—N5	28.14 (6)
C31—C30—C29	121.1 (3)	O5—Sc1—N5	27.78 (6)
C31—C30—H20	119.4	N6—Sc1—N5	92.81 (7)
C29—C30—H20	119.4	O2—Sc1—Sc2	38.95 (5)
C30—C31—C32	121.0 (3)	O1—Sc1—Sc2	35.69 (5)
C30—C31—H21	119.5	O9—Sc1—Sc2	123.21 (5)
C32—C31—H21	119.5	N1—Sc1—Sc2	100.71 (6)
C33—C32—C31	119.3 (3)	N2—Sc1—Sc2	97.72 (6)
C33—C32—H22	120.3	O8—Sc1—Sc2	176.27 (6)
C31—C32—H22	120.3	O6—Sc1—Sc2	80.73 (5)
C32—C33—C28	121.3 (3)	O5—Sc1—Sc2	107.26 (5)
C32—C33—H23	119.4	N6—Sc1—Sc2	151.21 (5)
C28—C33—H23	119.4	N5—Sc1—Sc2	90.74 (5)
N4—C34—C35	125.3 (3)	O4—Sc2—O3	89.96 (8)
N4—C34—H24	117.3	O4—Sc2—O1	161.16 (8)
C35—C34—H24	117.3	O3—Sc2—O1	103.19 (7)
C40—C35—C36	119.0 (3)	O4—Sc2—O11	85.82 (8)
C40—C35—C34	118.0 (3)	O3—Sc2—O11	78.90 (8)
C36—C35—C34	122.9 (2)	O1—Sc2—O11	83.59 (7)
O4—C36—C37	120.2 (3)	O4—Sc2—N4	78.40 (8)
O4—C36—C35	121.1 (2)	O3—Sc2—N4	129.49 (8)
C37—C36—C35	118.7 (2)	O1—Sc2—N4	102.50 (7)
C38—C37—C36	121.2 (3)	O11—Sc2—N4	146.80 (8)
C38—C37—H25	119.4	O4—Sc2—N3	119.10 (8)
C36—C37—H25	119.4	O3—Sc2—N3	76.53 (8)
C37—C38—C39	120.0 (3)	O1—Sc2—N3	77.63 (7)
C37—C38—H26	120.0	O11—Sc2—N3	144.55 (8)
C39—C38—H26	120.0	N4—Sc2—N3	67.60 (8)
C40—C39—C38	119.9 (3)	O4—Sc2—O2	94.97 (7)
C40—C39—H27	120.0	O3—Sc2—O2	153.38 (8)
C38—C39—H27	120.0	O1—Sc2—O2	67.28 (6)
C39—C40—C35	121.2 (3)	O11—Sc2—O2	75.40 (7)
C39—C40—H28	119.4	N4—Sc2—O2	77.07 (7)
C35—C40—H28	119.4	N3—Sc2—O2	122.46 (7)
O11—C41—C42	111.6 (3)	O4—Sc2—Sc1	128.53 (6)
O11—C41—H31	109.3	O3—Sc2—Sc1	133.60 (6)
C42—C41—H31	109.3	O1—Sc2—Sc1	33.78 (5)
O11—C41—H30	109.3	O11—Sc2—Sc1	79.39 (5)
C42—C41—H30	109.3	N4—Sc2—Sc1	87.84 (6)
H31—C41—H30	108.0	N3—Sc2—Sc1	99.75 (6)
C41—C42—H32	109.5	O2—Sc2—Sc1	33.61 (4)
			
C6—C1—C2—C3	−2.7 (4)	C27—C28—C33—C32	179.2 (3)
N1—C1—C2—C3	175.8 (3)	N4—C34—C35—C40	−174.3 (3)
C6—C1—C2—N2	179.2 (3)	N4—C34—C35—C36	8.9 (5)
N1—C1—C2—N2	−2.3 (4)	C40—C35—C36—O4	179.4 (3)
C1—C2—C3—C4	0.0 (5)	C34—C35—C36—O4	−3.9 (4)
N2—C2—C3—C4	177.9 (3)	C40—C35—C36—C37	−1.0 (4)
C2—C3—C4—C5	1.4 (5)	C34—C35—C36—C37	175.7 (3)
C3—C4—C5—C6	0.0 (5)	O4—C36—C37—C38	−178.2 (3)
C4—C5—C6—C1	−2.7 (5)	C35—C36—C37—C38	2.2 (5)
C2—C1—C6—C5	4.1 (5)	C36—C37—C38—C39	−1.8 (5)
N1—C1—C6—C5	−174.3 (3)	C37—C38—C39—C40	0.1 (5)
N1—C7—C8—C9	−23.9 (5)	C38—C39—C40—C35	1.1 (5)
N1—C7—C8—C13	160.8 (3)	C36—C35—C40—C39	−0.6 (5)
C13—C8—C9—O1	177.9 (3)	C34—C35—C40—C39	−177.5 (3)
C7—C8—C9—O1	2.7 (4)	C8—C7—N1—C1	174.4 (3)
C13—C8—C9—C10	−1.8 (4)	C8—C7—N1—Sc1	−2.5 (4)
C7—C8—C9—C10	−177.0 (3)	C6—C1—N1—C7	24.5 (4)
O1—C9—C10—C11	−177.2 (3)	C2—C1—N1—C7	−153.9 (3)
C8—C9—C10—C11	2.4 (4)	C6—C1—N1—Sc1	−158.2 (2)
C9—C10—C11—C12	−1.9 (5)	C2—C1—N1—Sc1	23.3 (3)
C10—C11—C12—C13	0.6 (5)	C15—C14—N2—C2	−179.4 (3)
C11—C12—C13—C8	0.1 (5)	C15—C14—N2—Sc1	2.8 (4)
C9—C8—C13—C12	0.6 (5)	C3—C2—N2—C14	−15.7 (4)
C7—C8—C13—C12	176.0 (3)	C1—C2—N2—C14	162.3 (3)
N2—C14—C15—C16	17.0 (5)	C3—C2—N2—Sc1	162.3 (2)
N2—C14—C15—C20	−165.8 (3)	C1—C2—N2—Sc1	−19.7 (3)
C20—C15—C16—O2	−174.7 (3)	C28—C27—N3—C21	−179.3 (3)
C14—C15—C16—O2	2.5 (4)	C28—C27—N3—Sc2	−1.6 (4)
C20—C15—C16—C17	2.9 (4)	C26—C21—N3—C27	30.0 (4)
C14—C15—C16—C17	−179.9 (3)	C22—C21—N3—C27	−150.6 (3)
O2—C16—C17—C18	175.1 (3)	C26—C21—N3—Sc2	−148.1 (2)
C15—C16—C17—C18	−2.4 (4)	C22—C21—N3—Sc2	31.3 (3)
C16—C17—C18—C19	1.0 (5)	C35—C34—N4—C22	−175.0 (3)
C17—C18—C19—C20	0.0 (5)	C35—C34—N4—Sc2	3.9 (4)
C18—C19—C20—C15	0.5 (5)	C23—C22—N4—C34	−34.8 (4)
C16—C15—C20—C19	−2.0 (5)	C21—C22—N4—C34	145.5 (3)
C14—C15—C20—C19	−179.4 (3)	C23—C22—N4—Sc2	146.2 (2)
C26—C21—C22—C23	0.3 (4)	C21—C22—N4—Sc2	−33.6 (3)
N3—C21—C22—C23	−179.2 (3)	C10—C9—O1—Sc1	−135.2 (2)
C26—C21—C22—N4	−179.9 (3)	C8—C9—O1—Sc1	45.2 (3)
N3—C21—C22—N4	0.6 (3)	C10—C9—O1—Sc2	47.0 (3)
C21—C22—C23—C24	0.5 (4)	C8—C9—O1—Sc2	−132.7 (2)
N4—C22—C23—C24	−179.2 (3)	C17—C16—O2—Sc1	138.4 (2)
C22—C23—C24—C25	−1.2 (5)	C15—C16—O2—Sc1	−44.1 (3)
C23—C24—C25—C26	1.1 (5)	C17—C16—O2—Sc2	−61.7 (3)
C24—C25—C26—C21	−0.2 (5)	C15—C16—O2—Sc2	115.8 (2)
C22—C21—C26—C25	−0.5 (4)	C30—C29—O3—Sc2	−176.3 (2)
N3—C21—C26—C25	178.9 (3)	C28—C29—O3—Sc2	4.2 (5)
N3—C27—C28—C33	179.4 (3)	C37—C36—O4—Sc2	161.4 (2)
N3—C27—C28—C29	−0.6 (5)	C35—C36—O4—Sc2	−19.0 (5)
C33—C28—C29—O3	180.0 (3)	O7—N5—O5—Sc1	−163.6 (2)
C27—C28—C29—O3	−0.1 (4)	O6—N5—O5—Sc1	15.2 (2)
C33—C28—C29—C30	0.5 (4)	O7—N5—O6—Sc1	163.5 (2)
C27—C28—C29—C30	−179.5 (3)	O5—N5—O6—Sc1	−15.4 (2)
O3—C29—C30—C31	−179.4 (3)	O10—N6—O8—Sc1	−179.5 (2)
C28—C29—C30—C31	0.0 (4)	O9—N6—O8—Sc1	0.1 (2)
C29—C30—C31—C32	−0.3 (5)	O10—N6—O9—Sc1	179.5 (2)
C30—C31—C32—C33	0.0 (5)	O8—N6—O9—Sc1	−0.1 (2)
C31—C32—C33—C28	0.6 (6)	C42—C41—O11—Sc2	87.3 (4)
C29—C28—C33—C32	−0.9 (5)		

### Hydrogen-bond geometry (Å, º)

**Table:**

*D*—H···*A*	*D*—H	H···*A*	*D*···*A*	*D*—H···*A*
O11—H29···O6	0.83 (2)	2.01 (2)	2.787 (3)	156 (3)
